# Risk Factors for Road Traffic Injuries among Different Road Users in the Gambia

**DOI:** 10.1155/2017/8612953

**Published:** 2017-04-23

**Authors:** Edrisa Sanyang, Corinne Peek-Asa, Paul Bass, Tracy L. Young, Babanding Daffeh, Laurence J. Fuortes

**Affiliations:** ^1^Department of Public & Environmental Health, School of Medicine & Allied Health Sciences, University of the Gambia, Brikama, Gambia; ^2^Injury Prevention and Research Center, College of Public Health, University of Iowa, Iowa City, IA, USA; ^3^Emergency Department, Serrekunda General Hospital, Kanifing, Gambia; ^4^Center for International Rural & Environmental Health, College of Public Health, University of Iowa, Iowa City, IA, USA

## Abstract

We identified risk factors for road traffic injuries among road users who received treatment at two major trauma hospitals in urban Gambia. The study includes pedestrians, bicyclists, motorcyclists, and drivers/passengers of cars and trucks. We examined distributions of injury by age, gender, collision vehicle types and vehicle category, and driver and environment factors. Two hundred and fifty-four patients were included in the study. Two-thirds were male and one-third female. Two-thirds (67%) of road traffic injuries involved pedestrians, bicyclists, and motorcyclists; and these were more common during weekdays (74%) than weekends. Nearly half (47%) of road traffic injuries involved pedestrians. One-third (34%) of injured patients were students (mean age of students was less than 14 years), more than half (51%) of whom were injured on the roadway as pedestrians. Head/skull injuries were common. Concussion/brain injuries were 3.5 times higher among pedestrians, bicyclists, and motorcyclists than vehicle occupants. Crashes involving pedestrians were more likely to involve young people (<25 years; aOR 6.36, 95% CI: 3.32–12.17) and involve being struck by a motor car (aOR 3.95, 95% CI: 2.09–7.47). Pedestrians contribute the largest proportion of hospitalizations in the Gambia. Young pedestrians are at particularly high risk. Prevention efforts should focus on not only vehicle and driver factors, but also protecting pedestrians, bicyclists, and motorcyclists.

## 1. Introduction

According to the World Health Organization (WHO), in 2013 more than 1.25 million people died from traffic crashes [1]. About 90% of the victims were from low middle income countries (LMICs) [1]. The road traffic related mortality rate in LMICs is not commensurate with the levels of motorization [1, 2]. Pedestrians, bicyclists, and motorcyclists are disproportionately represented and account for 49% of all traffic deaths [1], constituting an important public health problem.

In the Gambia, the national roadway infrastructure has improved since 2008 because of a European Union grant for construction of roads. The country has about 4,000 km of road network, with 1,800 km recognized as primary roads [3]. These roads connect major towns in the south bank and most of the north bank regions of the country, which is divided by a river [3]. In 2013, the World Economic Forum ranked Gambian roadway quality as 51st out of 140 countries [4]. However, roadway improvement has focused on high-speed national roads between towns, while infrastructure for local roads has lagged [3]. The road infrastructure development has led to increases in the transportation of goods and services with improved access and speed, while safety has not been a high priority. With sustained economic growth, urbanization, and overall social development, especially in the middle class, the level of motorization has increased. The number of registered vehicles increased from 17,416 in 2009 to 54,471 in 2013 for a population of 1.8 million [1].

Sustained economic growth is a leading factor in increasing motorization predominantly due to increases in per capita income and urbanization [2]. The growth of motorization generally outpaces the development of roadway safety infrastructure [2]. With increased suburban sprawl, developing and maintaining the infrastructure necessary to encourage road sharing among different road users (e.g., safety signs and road marks) becomes a challenge. Increase in per capita income (or increases in the middle class population) also comes with increases in private and commercial ownership of vehicles, therefore increasing the volume of traffic [2]. Injuries to pedestrians, bicyclists, and motorcyclists become inevitable without an appropriate safety infrastructure. Recovery from injury is further challenged for individuals who are injured in countries with underdeveloped trauma system. The Gambia does not have a national emergency ambulance service or an emergency number to call when an injury event occurs. There is only one neurosurgeon for the entire country and the second biggest trauma hospital (Serrekunda General Hospital) does not have an orthopedic unit. Lack of these essential services in lower level hospitals and other trauma hospitals adds pressure on the main referral hospital, Edward Francis Small Teaching Hospital. This hospital has six operating rooms with annual surgical admissions of about 5000 [5] but lacks capacity to perform equipment-intensive procedures. Patients with severe traumatic brain injuries needing equipment-intensive procedures are sent out of country or for overseas treatment.

Rapid motorization and urbanization have contributed to traffic congestion in the Greater Banjul Area, and the majority of vehicles include cars and four-wheel light vehicles. Poor driving culture, such as failing to yield to pedestrians and lack of pedestrian crossings, put pedestrians, bicyclists, and motorcyclists at risk of road traffic injuries [6]. No prior studies have assessed risks of injury associated with different road users in the Gambia. The goal of this study is to identify differences in road user, collision, vehicle, and driver factors among individuals hospitalized with a road traffic injury. We hypothesized that both crash outcome and driver characteristics differ by the road user type, which we categorized as pedestrians, bicyclists, motorcyclists, and vehicle occupants. Data were collected from the two trauma hospitals in the Gambia.

## 2. Methods

### 2.1. Study Sites and Trauma Registries

**Figure 1:**
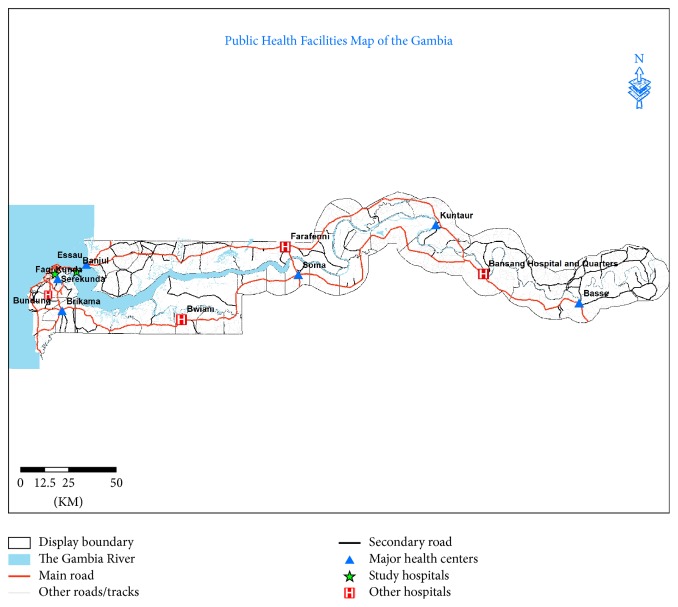
Map of the Gambia showing location of study hospitals.

This study was conducted with prospectively collected data from admitted road traffic trauma patients in two major trauma hospitals in the Gambia: Edward Francis Small Teaching Hospital (EFSTH) and Serrekunda General Hospital (SGH) (Figure 1). The trauma registries were initiated by the University of the Gambia in partnership with these hospitals. It was implemented to examine the feasibility of establishing trauma system with focus on type and nature of injuries presented to the two major trauma hospitals in urban Gambia. The registries capture data about all severely injured patients seen at the two hospitals requiring hospitalization across the country but also provide routine healthcare needs (including trauma) of Kanifing Municipal and Banjul City Councils (population, 413,397) [7]. The two councils form the catchment area of the registry hospitals which makes up about 22% of the population in the Gambia.

In the road traffic injuries (RTI) trauma registries, a case is defined as a traffic injury if the patient who visits either of the two hospitals presents with an injury that meets the following conditions: (1) injured patient is a motor vehicle occupant, motorcyclist, bicyclist, pedestrian, or occupant or driver of animal-driven cart; (2) incident leading to injury occurred on a public or private highway, street, or road; and (3) injured patient is admitted to the study hospitals for more than 24 hours. All individuals who met the above criteria and provided consent were included in the study.

### 2.2. Study Population and Data Collection

Patients admitted to one of the study hospitals for treatment of an injury that occurred on a roadway involving traffic were eligible for study inclusion. Admitting physicians determined eligibility based on the cause of injury as reported by the patient or caregiver. There were 262 admitted cases from March 1, 2014, to March 31, 2016. Eight patients with incomplete data were excluded from this analysis, leaving a study population of 254 patients. Treating physicians used a 29-item questionnaire to collect data at the time of admission. Data about risks contributing to crashes and injuries were collected from patients. Where patients were not mentally alert or could not answer the questions, an adult family member was accepted and interviewed. The questionnaire described road user type, body parts injured, nature of injury, date and time of injury, environmental characteristics, and vehicle and driver factors contributing to a crash.

### 2.3. Variables

Road user type was the primary dependent variable. Road users were categorized as pedestrians, bicyclists, motorcyclists, animal-driven cart passengers, and drivers and passengers of cars and trucks.

Our main independent variables included personal, environmental, vehicle, and driver factors. Personal ones included age, gender, and occupation. Occupation was categorized as professional (including civil servant, business, and health worker), skilled (included skilled worker, driver, and security officer), unskilled (farmer), student, and others (including housewife and those who support commercial drivers collecting money from passengers). Environmental features included day of week, time of day, time of year, and driver visibility of the roadway environment. Vehicle factors included brake failure, burst tires, collision vehicle type (including motor car, van, bus, truck, and motorcycle), and collision vehicle category (included private or commercial cars). Motor cars are four-passenger vehicles or light four-wheel drives and can be both commercial or private cars. Commercial cars are those vehicles available to public use with cost and range from four passengers to 30-seater minibuses. Collision vehicle type and category apply to the vehicle that was involved in the crash with the injured party. Driver factors included age, gender, drug or alcohol use, and speeding. Primary nature of injury and primary body parts injured were collected on the injured patients. Primary nature of injury was categorized as soft tissue (which includes open wound, abrasion, and contusion); fracture; dislocation (including sprain and strain); concussion/brain injury; and others (including foreign body, burns and scalds, injury to muscle/tendons/blood vessels/nerves, injury to internal organs, crush injury, amputation, suffocation, and multiple injuries). Body parts injured were categorized as head/skull; face and neck; thoracic area/lumbar spine/abdominal area; lower extremity/pelvis/hip; upper extremity; multiple body parts; and others/unknown.

### 2.4. Analysis

Cross-tabulations of road user type were examined by individual, vehicle, and environmental factors. The primary nature of injury and the body part injured were examined to identify the most prevalent injury profiles for each road user type. Odd ratios identifying the association of covariates (age, day of week, collision vehicle type, vehicle category, speed, and poor visibility) for each type of road user compared with all other road users were calculated. All covariates were examined for inclusion in the logistic regression model using a forward selection method with a specified level for entry set at *p* < 0.20. All analyses were performed using SAS 9.4 (SAS Institute, Inc., Cary, NC, USA).

### 2.5. Ethical Approval

Ethical approval for this study was obtained from the joint Gambia Government/Medical Research Council (MRC) Ethics Committee and the University of Iowa Institutional Review Board. Data collection was conducted in accordance with the Helsinki Declaration.

## 3. Results

### 3.1. Crash Characteristics at the Personal Level

Among the 6,491 injured patients treated at Edward Francis Small Teaching Hospital and Serrekunda General Hospital (from March 1, 2014, to March 31, 2016) 2,196 were road traffic related (34%). Of these, 262 (12%) were admitted to one of the two hospitals, and 254 patients comprised our study population, after we excluded eight with incomplete data.

Two-thirds (67%) of the patients hospitalized with road traffic injuries (RTI) involved pedestrians (47%) and bicyclists/motorcyclists (21%) (Table 1). Of all RTI, more than two-thirds (68%) were among males and 32% were among females. Over 94% of bicycle/motorcycle injuries were among males; and 58% of the vehicle-occupant injuries were sustained by males. More than half (52%) of the RTI involved those under the age of 25 years. For both genders, there were more RTI hospitalizations for pedestrians and vehicle occupants (driver or passenger) than bicyclists and motorcyclists.

About one-third (34%) of all injured patients were students (mean age of students is less than 14 years). The proportions of students injured as bicyclists/motorcyclists (24%) and in-vehicle occupants (15.8%) were lower compared to those injured as pedestrians (51%; Table 1). Almost half (47%) of patients injured as vehicle occupants and 54% of those injured as bicyclists/motorcyclists were professional or skilled workers.

### 3.2. Crash Characteristics, Other

The majority (71%) of crashes involving all forms of road users occurred between 6 AM and 5:59 PM. Crashes involving pedestrian and bicyclists/motorcyclists were frequent between 12 PM and 5:59 PM. Almost three-fourths (74%) of crashes occurred on weekdays and more commonly (73%) during the dry season.

Overall, the majority (77%) of all crashes involving pedestrians were caused by either motor cars or vans (Table 2). The majority (73%) of bicyclist/motorcyclist injuries occurred due to a motorcar colliding with a bicycle/motorcycle or bicycles/motorcycles colliding with each other or persons falling off by crash. Trucks/truck trailers hitting pedestrians were not frequently reported.

Speeding (79%) was frequently reported as contributing to crashes involving all road users. The speeding contributed to even more crashes involving pedestrians (82%) than among bicyclists/motorcyclists (77%) and vehicle occupants (76%). Roadway visibility (28%) was a contributing cause of crashes, especially among bicyclists/motorcyclists (35%) compared to vehicle occupant injuries (24%). Drug or alcohol use (2%), burst tires (8%), and brake failures (18%) were less frequently reported.

### 3.3. Injury Characteristics by Road User Type

Lower extremity/pelvis/hip were the most commonly injured body parts among all road user types, with soft tissue injuries being the most common nature of injury (Table 3). Concussion/brain injury was 3.5 times higher among pedestrians, bicyclists, and motorcyclists than vehicle occupants. The proportion of multiple injuries among vehicle occupants (81%) and bicyclists/motorcyclists (79%) was slightly higher than among pedestrians (75%).

Crashes involving pedestrians were more likely to be among young people (<25 years; aOR 6.36, 95% CI: 3.32–12.17) compared to those 25+ years and to involve being struck by a motor car (aOR 3.95, 95% CI: 2.09–7.47) compared to all other vehicle types (Table 4). Pedestrian injuries were less likely to involve commercial vehicles (aOR 0.43, 95% CI: 0.22–0.82). Although not statistically significant, the data suggests crashes involving bicyclists/motorcyclists were more likely to occur under poor roadway visibility (aOR 1.9, 95% CI: 0.89–3.94) and were much less likely to involve young people (aOR 0.24, 95% CI: 0.1–0.52). The data also suggests vehicle occupants (compared to all other road users) were 1.8 times as likely to be involved in a weekend crash (aOR 1.8, 95% CI: 1.02–3.3) or a crash involving a commercial vehicle (aOR 3.7, 95% CI: 1.81–7.6).

## 4. Discussion

Injuries to pedestrians, bicyclists, and motorcyclists are an important public health burden in the Gambia. The majority of new roads in the country's infrastructure has been developed via European Union funds. With these funds, 35.4% [8] of the roadways were paved, helping the Gambia lead the West African Subregion in road transport (quality index, 41.6) [9]. Nevertheless, roadway improvement projects have focused on promoting internal circulation of goods and services as well as reexporting trade to neighboring countries [10] rather than local roads used more frequently by residents. This study identified that collision, vehicle, and driver variables were more strongly associated with injuries to pedestrians, bicyclists, and motorcyclists than vehicle occupants. One major limitation of the study is that road user exposure could not be determined because information on traffic and pedestrian volume was not available.

Crashes involving pedestrians, compared to all other road users, were more likely to be among young people (<25 years). More than half (52%) of the RTI patients were under 25 years, and a greater proportion of them (34%) were students (mean age is less than 14 years). In a similar study in Nigeria, the proportion of students injured as pedestrians (20%) was less than reported here [11]. Moreover, when compared to vehicle occupants (16%) who were injured, the proportion of students (mean age is less than 14 years) injured as pedestrians (51%) was greater than that of all other groups. In a similar study in Australia, the opposite was found, with student involvement in crashes being rare [12]. The differences in these findings could be due to levels of sociocultural factors, road use pattern, and road infrastructure development in the various countries and differing exposures for road users by age. Whatever the situation, it is well known that young people frequently take risks related to road safety by disobeying traffic rules such as darting into the roadway and jaywalking [13–15]. Child safety in the roadway is a major public health concern in low-income countries. Solutions are likely to have higher impact in promoting child safety than trying to modify child behavior or roadway exposure. Some intervention strategies include increasing pedestrians' visibility when crossing or traveling along the street, traffic-slowing measures, and reducing pedestrian exposure to traffic by designing roadways to accommodate them.

Additionally, our findings indicate that crashes involving pedestrians compared to all other road users were more likely to involve speeding by motor vehicles. Although the Gambia has speed limits for highways (World Health Organization, 2015), there are no speed limits for local roads [1]. Enforcement of speed limits is essential for making them truly effective [1]. According to the WHO, the enforcement of speed laws in the Gambia was assessed as 5 on a scale of 10 [1] which implies weak enforcement. The association between vehicle speed and pedestrian injury has been well described. In effect, reducing vehicle speeds would have reduced the pedestrian injury burden by either eliminating some crashes all together, and/or reducing injury severity [16, 17].

Results are consistent with established risk factors for crashes involving bicyclists and motorcyclists [18–20]. Crashes involving bicyclists/motorcyclists compared to all other road users were more likely to be due to driver's poor visibility of the roadway environment. In a similar hospital based study on risk for crashes in Nigeria and Kenya, results also indicate that poor driver visibility of pedestrians compared to other road users is an important factor [11, 21, 22]. The mix of motorized and nonmotorized traffic, together with poor street lighting, increased the risk of pedestrians, bicyclists, and motorcyclists not being seen. The unsafe situation can be exacerbated when pedestrians, bicyclists, and motorcyclists do not use low cost interventions such as reflective equipment.

Furthermore, our findings indicate that almost three-fourths (73%) of bicyclist/motorcyclist injuries occurred due to bicycle/motorcycle colliding with a motor vehicle, bicycles/motorcycles colliding with each other, or bicycle/motorcycle passengers falling off on their own. Although, between 2009 and 2013, the total number of registered vehicles in the Gambia increased only threefold, motorized, two/three-wheeled, registered vehicles increased almost eightfold [1, 23]. Thus policies, roadway designs, and safety culture approaches need to focus on bicyclists and motorcyclists so that injury rates do not increase with increasing kilometers traveled.

Crashes involving vehicle occupants when compared to all other road users were more likely to involve a commercial vehicle and to occur on weekends. This study found crashes involving pedestrians, bicyclists, and motorcyclists mostly occur during weekdays. About 60% of the Gambia's population lives in urban areas [7], while most of the government and other agency offices, service, and commerce are located in city centers. Although average annual daily traffic and pedestrian volume data are not available, this urban concentration creates a heavy commuting burden throughout most of the weekdays. Since most individuals do not use cars, the community traffic burden mix is complex, involving pedestrians, bicyclists, motorcyclists, and a mix of private and commercial vehicles.

Our results indicate that concussion was more common among pedestrians, bicyclists, and motorcyclists than vehicle occupants. This is consistent with similar studies in other regions [24, 25]. Nevertheless, the proportion of multiple injuries was higher among vehicle occupants, bicyclists, and motorcyclists than pedestrians. The high incidence of concussions among pedestrians, who are often poorer compared to other road users, can increase the burden of trauma in families and the country, especially in a situation such as the Gambia where no prehospital care system exists. The country does not have a national ambulance system or a functional emergency number to call in case of a traumatic injury event. The Gambia Fire and Rescue Services do have some rescue capacity, but the public is not well aware of this function and the services are not utilized. When called, responders decide on the healthcare facility destination for treatment of trauma patients on their own, without information about treatment capacity or triage protocols. The implications are that many of the more seriously injured patients are not transported to the definitive care hospitals. Moreover, the entire country completely lacks emergency physicians and nurses with specialized training in emergency care [23]. Furthermore, little legislation addresses safety for pedestrians, bicycles, and two-wheeled motorized vehicles, although these are the road users most frequently injured in the Gambia.

Currently, no agency in the Gambia is charged to lead traffic safety efforts. The National Road Authority was set up by an act of parliament with the mandates for road construction, rehabilitation, and promotion of safer roads. However, the safety component is not developed. The Ministry of Health and Social Welfare subsumed the uncoordinated road safety efforts under the noncommunicable diseases prevention and control mandate. One contributing factor for the lack of safety as a priority is the lack of data that can identify priority areas to guide prevention programs. Now that risks for road traffic injuries in the Gambia are identified, the government of the Gambia can prioritize consolidating and advancing road safety. The first step is to stimulate interest to bring partners in road safety together through creation of road safety policies and strategies and establish the national road safety commission.

### 4.1. Limitations

Trauma registry data are well recognized for underrepresenting all injury events and disproportionately represent patients with more severe injuries. Thus, the sample analyzed is not likely to be fully representative of the spectrum of road traffic injury severity.

This study was also limited to the data available in the trauma registries at EFSTH and SGH. Data about contributors to crashes were collected from patients, and where patients were not mentally alert and could not answer questions, answers were retrieved from adult relatives. It is possible that some of these variables may be biased and could have inconsistent reporting. Patient self-reporting risks, such as drunk-driving and use of illicit drugs, could be low due to legal implications. Crash characteristics are related to roadway exposure, but we lacked information about roadway exposure, introducing inconsistencies. Despite these limitations, the results from this study encourage further investigation of collision vehicle and driver factors, including exposure data from the roadway environment.

## 5. Conclusions

In general, prevention strategies in the Gambia have been mainly implemented for vehicle occupant, whereas little attention has been paid to pedestrian, bicyclist, and motorcyclist safety on the road. Major interventions that were recently implemented in the Gambia included the introduction of obligatory front seat belts and banning cell phone use while driving [1, 23]. This study does not evaluate the effectiveness of these interventions, but they could contribute in general to a decrease in injuries among vehicle occupants. However, injuries to pedestrians, bicyclists, and motorcyclists continue to be a major public health concern because of the lack of attention to safety in urban development, roadway planning, and policy implementation [1, 23].

The results reveal associations between different road user categories, age, occupation, and injury profiles. RTI patients were frequently young adults (<25 years) and mostly students (mean age is less than 14 years) who were consistently identified by WHO and the United Nations as an at-risk group for road traffic crashes (as indicated in the recently crafted Sustainable Development Goals). Recognition of these features would be useful in designing effective prevention strategies including attaining Sustainable Development Goals.

## Figures and Tables

**Table 1:** Patient demographics by road user type^1^.

Factors	Total^2^		Pedestrian		Bicyclist/motorcyclist		In-vehicle occupant	
	*N*	(%)	*N*	(%)	*N*	(%)	*N*	(%)
*Total (N, %)*	254	(100.0)	118	(46.5)	52	(20.5)	79	(31.1)

Age (years)								
<25	127	(52.0)	85	(73.3)	15	(29.4)	26	(34.7)
>25	117	(48.0)	31	(26.7)	36	(70.6)	49	(65.3)
Gender								
Male	169	(67.6)	74	(62.7)	49	(94.2)	45	(57.7)
Female	81	(32.4)	44	(37.3)	3	(5.8)	33	(42.3)
Occupation								
Skilled	42	(17.4)	12	(10.5)	12	(24.0)	18	(23.7)
Professional	44	(18.2)	11	(9.6)	15	(30.0)	18	(23.7)
Unskilled	14	(5.8)	3	(2.6)	5	(10.0)	6	(7.9)
Student	82	(33.9)	58	(50.9)	12	(24.0)	12	(15.8)
Other	56	(23.1)	30	(26.3)	6	(12.0)	18	(23.7)
Missing	4	(1.7)	0	(0.0)	0	(0.0)	4	(5.3)

^1^Road user type: “Other” was not depicted as separate column in table due to small number (*n* = 2; 0.78% of total) yet is included in total column.

^2^Numbers may not add to 254 due to missing data.

**Table 2:** Crash/environment and driver/vehicle factors by road user type^1^.

Factors	Total^2^		Pedestrian		Bicyclist/motorcyclist		In-vehicle occupant	
	*N*	(%)	*N*	(%)	*N*	(%)	*N*	(%)
Total (*N*, %)	254	(100.0)	118	(46.5)	52	(20.5)	79	(31.1)

*Environmental factors*								
Day of week								
Weekend	65	(25.9)	23	(19.5)	15	(28.8)	27	(34.2)
Weekday	186	(74.1)	95	(80.5)	37	(71.2)	52	(65.8)
Time of day								
12:00–5:59 AM	14	(5.6)	5	(4.2)	2	(3.8)	7	(8.9)
6:00–11:59 AM	77	(30.7)	32	(27.1)	13	(25.0)	30	(38.0)
12:00–5:59 PM	100	(39.8)	56	(47.5)	24	(46.2)	20	(25.3)
6:00–11:59 PM	60	(23.9)	25	(21.2)	13	(25.0)	22	(27.8)
Season								
Dry	183	(72.9)	75	(63.6)	43	(82.7)	64	(81.0)
Rainy	68	(27.1)	43	(36.4)	9	(17.3)	15	(19.0)
Poor visibility								
No	182	(72.5)	86	(72.9)	34	(65.4)	60	(75.9)
Yes	69	(27.5)	32	(27.1)	18	(34.6)	19	(24.1)
Collision vehicle type								
Motor car	112	(44.6)	74	(62.7)	18	(34.6)	19	(24.1)
Van	36	(14.3)	17	(14.4)	1	(1.9)	18	(22.8)
Mini bus	25	(10.0)	5	(4.2)	1	(1.9)	18	(22.8)
Pickup	15	(6.0)	4	(3.4)	3	(5.8)	8	(10.1)
Truck/truck trailer	21	(8.4)	3	(2.5)	2	(3.8)	16	(20.3)
Motorcycle	31	(12.4)	11	(9.3)	20	(38.5)	0	(0.0)
Other/unknown	11	(4.4)	4	(3.4)	7	(13.5)	0	(0.0)
Collision vehicle category								
Private	101	(41.1)	60	(52.2)	23	(46.0)	18	(22.8)
Commercial	125	(50.8)	50	(43.5)	18	(36.0)	55	(69.6)
Other/unknown	20	(8.1)	5	(4.3)	9	(18.0)	6	(7.6)

*Crash characteristics*								
Drug/alcohol influence								
No	246	(98.0)	118	(100.0)	51	(98.1)	75	(94.9)
Yes	5	(2.0)	0	(0.0)	1	(1.9)	4	(5.1)
Speeding								
No	52	(20.7)	21	(17.8)	12	(23.1)	19	(24.1)
Yes	199	(79.3)	97	(82.2)	40	(76.9)	60	(75.9)
Brake failure								
No	206	(82.1)	92	(78.0)	46	(88.5)	68	(86.1)
Yes	45	(17.9)	26	(22.0)	6	(11.5)	11	(13.9)
Burst tire								
No	232	(92.4)	118	(100.0)	50	(96.2)	63	(79.7)
Yes	19	(7.6)	0	(0.0)	2	(3.8)	16	(20.3)

^1^Road user type: “Other” was not depicted as separate column in table due to small number (*n* = 2; 0.78% of total) yet is included in total column.

^2^Numbers may not add to 254 due to missing data.

**Table 3:** Injury characteristics by road user type.

Factors	Total^1^		Pedestrian		Bicyclist/motorcyclist		In-vehicle occupant	
	*N*	(%)	*N*	(%)	*N*	(%)	*N*	(%)
Total (*N*, %)^2^	254	(100.0)	118	(46.5)	52	(20.5)	79	(31.1)

Primary nature of injury^3^								
Soft tissue (open wound/abrasion/contusion)	94	(37.5)	42	(35.6)	23	(44.2)	29	(36.7)
Fracture	88	(35.1)	43	(36.4)	19	(36.5)	26	(32.9)
Dislocation/sprain/strain	13	(5.2)	4	(3.4)	2	(3.8)	7	(8.9)
Concussion/brain injury	42	(16.7)	26	(22.0)	6	(11.5)	9	(11.4)
Other/unknown	14	(5.6)	3	(2.5)	2	(3.8)	8	(10.1)
Primary body part								
Head/skull	74	(29.5)	46	(39.0)	10	(19.2)	17	(21.5)
Face/neck	33	(13.1)	9	(7.6)	11	(21.2)	13	(16.5)
Thorax/lumbar spine/abdomen	10	(4.0)	5	(4.2)	1	(1.9)	4	(5.1)
Lower extremity/pelvis/hip	96	(38.2)	50	(42.4)	21	(40.4)	24	(30.4)
Upper extremity	29	(11.6)	5	(4.2)	7	(13.5)	17	(21.5)
Multiple body parts	8	(3.2)	3	(2.5)	2	(3.8)	3	(3.8)
Other/unknown	1	(0.4)	0	(0.0)	0	(0.0)	1	(1.3)
Multiple injuries^3^								
Yes	55	(21.9)	29	(24.6)	11	(21.2)	15	(19.0)
No	196	(78.1)	89	(75.4)	41	(78.8)	64	(81.0)

^1^Numbers may not add to 254 due to missing data.

^2^Road user type: “Other” was not depicted as separate column in table due to small number (*n* = 5; 2% of total) yet is included in “Total” column.

^3^Primary nature of injury: these variables are not mutually exclusive but are the most prevalent injury reported by the patients.

**Table 4:** Predictors of crash/injury by road user type^1,2^.

Covariates	Total^3^	Pedestrians versus all other road users				Motorcyclist/bicyclists versus all other road users				Vehicle occupants versus all other road users			
		Crude		Adjusted		Crude		Adjusted		Crude		Adjusted	
		OR	95% CI	OR	95% CI	OR	95% CI	OR	95% CI	OR	95% CI	OR	95% CI
Age (years)													
<25	132	5.38	3.12–9.26	6.36	3.32–12.17	0.29	0.15–0.57	0.24	0.1–0.52	0.38	0.22–0.67	0.36	0.19–0.69
25+	118	Ref	NA	Ref	NA	Ref	NA	Ref	NA	Ref	NA	Ref	NA
Day of week													
Weekday	133	Ref	NA	Ref	NA	Ref	NA	NA^3^	NA^3^	Ref	NA	NA^3^	NA^3^
Weekend	118	0.53	0.29–0.94	0.34	0.16–0.71	1.21	0.61–2.39	NA^3^	NA^3^	1.8	1.02–3.3	NA^3^	NA^3^
Collision vehicle type													
Motor car	118	4.2	2.48–7.14	3.95	2.09–7.47	0.6	0.31–1.12	NA^3^	NA^3^	3.6	1.95–6.74	0.24	0.12–0.47
Other	133	Ref	NA	Ref	NA	Ref	NA	NA^3^	NA^3^	Ref	NA	Ref	NA
Collision vehicle category^4^													
Commercial	110	0.46	0.27–0.78	0.43	0.22–0.82	NA^3^	NA^3^	0.55	0.27–1.12	NA^3^	NA^3^	3.7	1.81–7.6
Private	116	Ref	NA	Ref	NA	NA^3^	NA^3^	Ref	NA	NA^3^	NA^3^	Ref	NA
Speeding													
Yes	118	1.4	0.75–2.61	NA^3^	NA^3^	0.84	0.4–1.74	NA^3^	NA^3^	0.75	0.34–1.42	NA^3^	NA^3^
No	133	Ref	NA	NA^3^	NA^3^	Ref	NA	NA^3^	NA^3^	Ref	NA	NA^3^	NA^3^
Poor visibility													
Yes	118	0.97	0.55–1.68	NA^3^	NA^3^	1.54	0.8–3.0	1.9	0.89–394	0.77	0.42–1.43	0.6	0.29–1.3
No	133	Ref	NA	NA^3^	NA^3^	Ref	NA	Ref	NA	Ref	NA	Ref	NA

^1^Road user type: “Other” was not depicted as separate column in table due to low number (*n* = 5; 2% of total) yet is included in “Total” column.

^2^Adjusting for all the variables in the table.

^3^Numbers may not add to 254 due to missing data.

^4^Did not meet the 0.2 significance level for entry into the model.
